# Supplementary material for: A Systematic Study of the Temperature Dependence of the Dielectric Function of GaSe Uniaxial Crystals from 27 to 300 K
Source: Nanomaterials (Basel). 2024 May 10;14(10):839. doi: 10.3390/nano14100839 (PMC11124486; doi:10.3390/nano14100839)

Supplementary Information for

Temperature dependence of the dielectric tensor of GaSe uniaxial crystal from 27 to 300 K

Long V. Le[1*], Tien-Thanh Nguyen[1], Xuan Au Nguyen[2], Do Duc Cuong[3], Thi Huong Nguyen[4,6], Van Quang Nguyen[5,6], Sunglae Cho[6], Young Dong Kim[2], and Tae Jung Kim[2*]

[1] Institute of Materials Science, Vietnam Academy of Science and Technology, Hanoi 100000, Vietnam;
[2] Department of Physics, Kyung Hee University, Seoul 02447, South Korea;
[3] Faculty of Physics and Engineering Physics, University of Science, VNU-HCM, Ho Chi Minh City, 70000, Vietnam;
[4] Department of Physics, Nha Trang University, Nha Trang 650000, Vietnam;
[5] Advanced process development team, ISAC research Inc., Techno2ro 340, Tabrip-dong, Yuseong-gu 34036, South Korea;
[6] Department of Physics and Energy Harvest Storage Research Center, University of Ulsan, Ulsan 44610, Republic of Korea;
*Correspondence: longlv@ims.vast.ac.vn (L.V.L.), tjkim@khu.ac.kr (T.J.K)

Figure Captions

Figure S1. EDX spectrum of the GaSe single crystal.

Figure S2. Out-of-plane Raman spectra of the GaSe single crystal with various polarization angles. 0° denotes the incident light polarized parallel to the c-axis.

Figure S3. Point-by-point fitting method used to remove surface roughness artifacts for the pseudodielectric functions along (a) the c-axis and (b) the a-axis of GaSe at 27 K. Data are given by dashed curves and fitted results by solid curves.

Figure S4. The graph shows the imaginary part ε_2 of the dielectric functions of GaSe. The solid lines represent the data from the present work. For comparison, digitized versions of previously reported data from Ref. [30] are also included.

Figure S5. Comparison of c-axis lineshape fitting assuming one and two critical points in the exciton region of the GaSe single crystal at 27 K.

Figure S6. The energy band structure of GaSe calculated before (solid curve) and after (dashed curve) bandgap correction using the mBJ method.

Figure S1

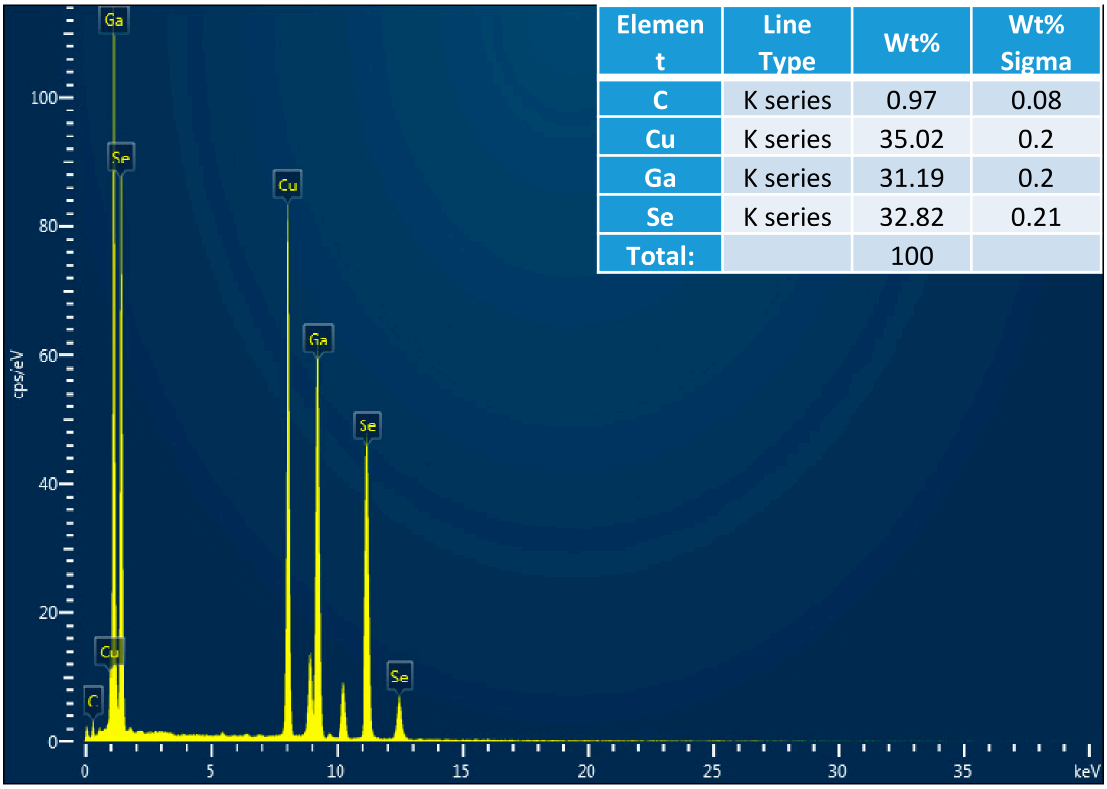

Element	Line Type	Wt%	Wt% Sigma
C	K series	0.97	0.08
Cu	K series	35.02	0.2
Ga	K series	31.19	0.2
Se	K series	32.82	0.21
Total:		100	

Figure S2

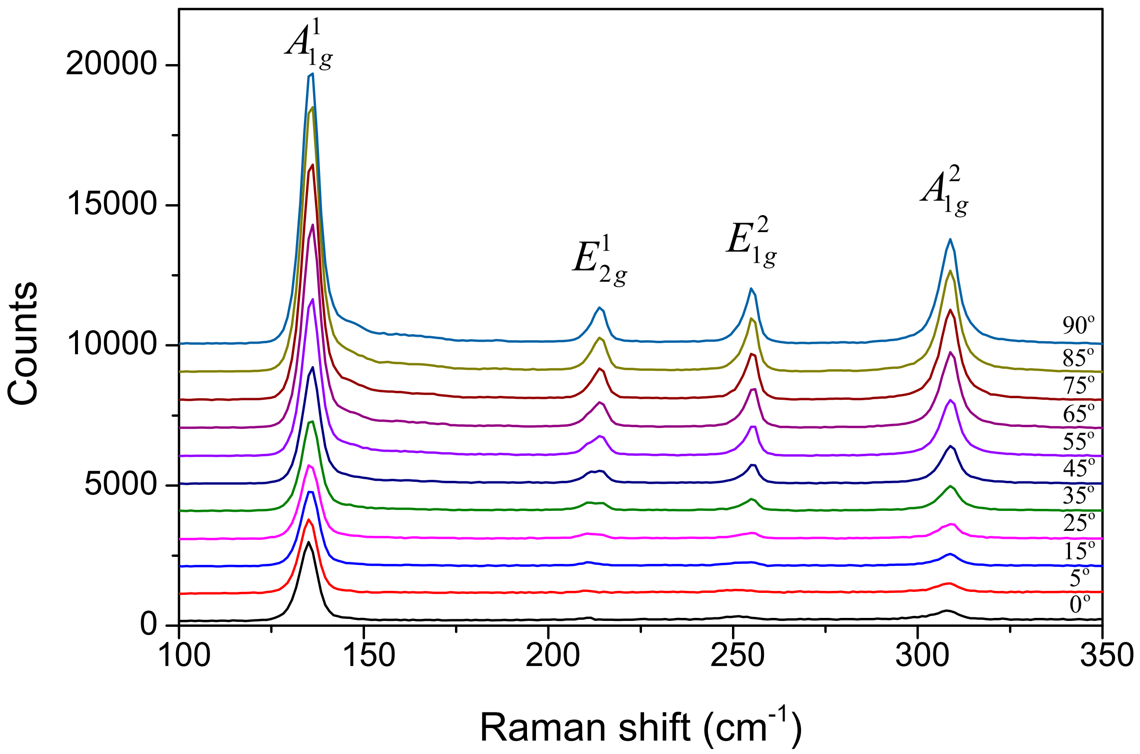

Counts
Raman shift (cm-1)
A^1_{1g}
E^1_{2g}
E^2_{1g}
A^2_{1g}
90°
85°
75°
65°
55°
45°
35°
25°
15°
5°
0°

Figure S3

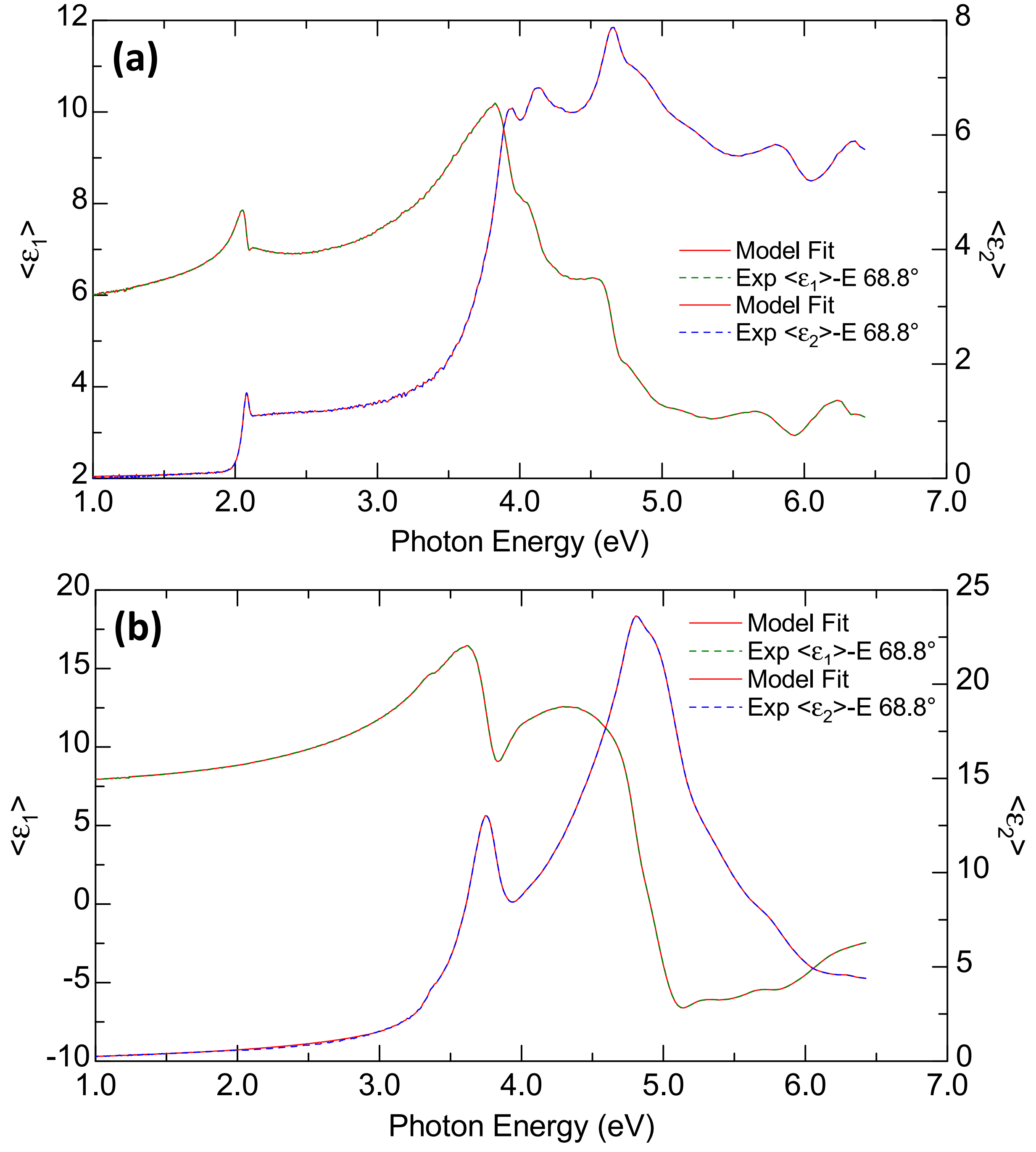

(a)
12
10
8
6
4
2
<ε1>
8
6
4
2
0
<ε2>
Model Fit
Exp <ε1>-E 68.8°
Model Fit
Exp <ε2>-E 68.8°
1.0
2.0
3.0
4.0
5.0
6.0
7.0
Photon Energy (eV)
(b)
20
15
10
5
0
-5
-10
<ε1>
25
20
15
10
5
0
<ε2>
Model Fit
Exp <ε1>-E 68.8°
Model Fit
Exp <ε2>-E 68.8°
1.0
2.0
3.0
4.0
5.0
6.0
7.0
Photon Energy (eV)

Figure S4

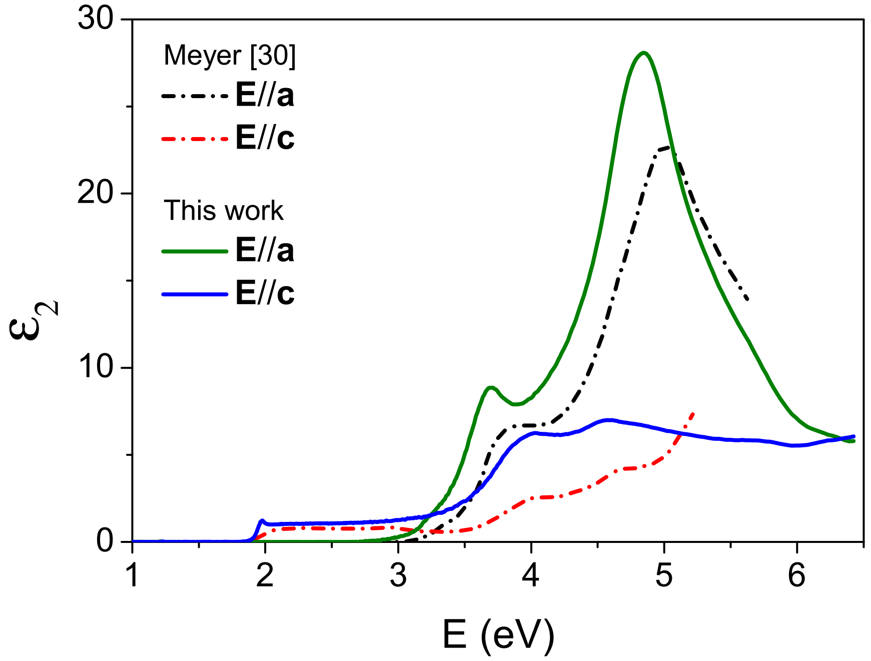

Figure S5

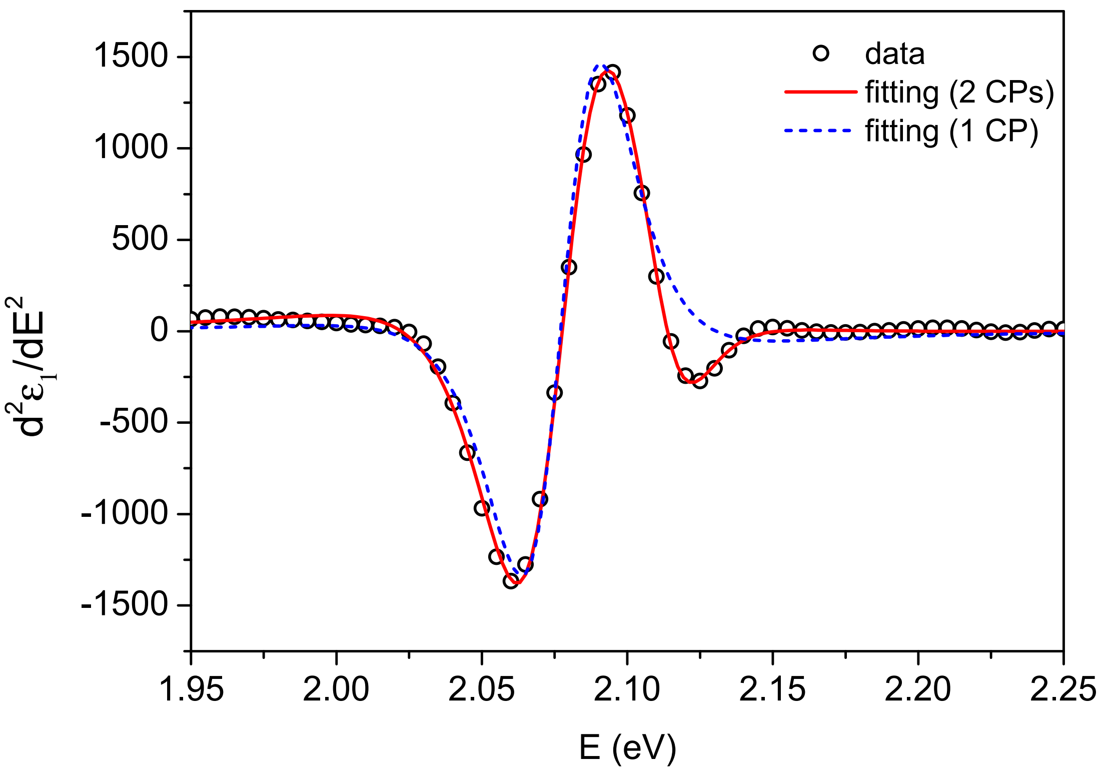

data
fitting (2 CPs)
fitting (1 CP)
1500
1000
500
0
-500
-1000
-1500
$d^2\varepsilon_1/dE^2$
1.95
2.00
2.05
2.10
2.15
2.20
2.25
E (eV)

Figure S6

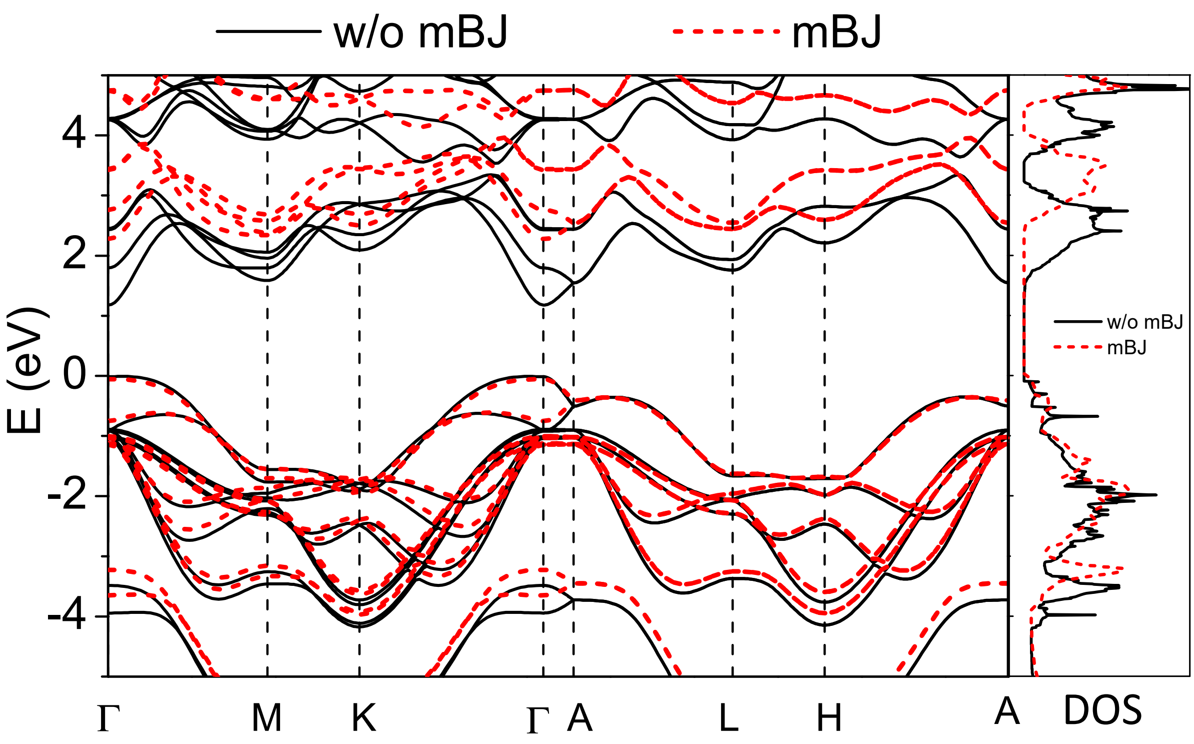

Supplement: Supplementary file 1 [file nanomaterials-14-00839-s001.zip › nanomaterials-2992154-supplementary.pdf]
